# Neoadjuvant short-course hyperfractionated accelerated radiotherapy (SC-HART) combined with S-1 for locally advanced rectal cancer

**DOI:** 10.1093/jrr/rrt058

**Published:** 2013-05-08

**Authors:** Hiroshi Doi, Naohito Beppu, Soichi Odawara, Masao Tanooka, Yasuhiro Takada, Yasue Niwa, Masayuki Fujiwara, Fumihiko Kimura, Hidenori Yanagi, Naoki Yamanaka, Norihiko Kamikonya, Shozo Hirota

**Affiliations:** 1 Department of Radiology, Hyogo College of Medicine, 1-1, Mukogawa-cho, Nishinomiya City, Hyogo, 663-8501 Japan; 2 Department of Surgery, Meiwa Hospital, 4-31 Agenaruo, Nishinomiya, Hyogo 663-8186, Japan

**Keywords:** rectal cancer, accelerated hyperfractionated radiotherapy, preoperative radiotherapy, short-course radiotherapy, radiotherapy, S-1

## Abstract

The purpose of this study was to examine the safety and feasibility of a novel protocol of neoadjuvant short-course hyperfractionated accelerated radiotherapy (SC-HART) combined with S-1 for locally advanced rectal cancer. A total of 56 patients with lower rectal cancer of cT3N1M0 (Stage III b) was treated with SC-HART followed by radical surgery, and were analyzed in the present study. SC-HART was performed with a dose of 2.5 Gy twice daily, with an interval of at least 6 hours between fractions, up to a total dose of 25 Gy (25 Gy in 10 fractions for 5 days) combined with S-1 for 10 days. Radical surgery was performed within three weeks following the end of the SC-HART. The median age was 64.6 (range, 39–85) years. The median follow-up term was 16.3 (range, 2–53) months. Of the 56 patients, 53 (94.4%) had no apparent adverse events before surgery; 55 (98.2%) completed the full course of neoadjuvant therapy, while one patient stopped chemotherapy because of Grade 3 gastrointestinal toxicity (CTCAE v.3). The sphincter preservation rate was 94.6%. Downstaging was observed in 45 patients (80.4%). Adjuvant chemotherapy was administered to 43 patients (76.8%). The local control rate, disease-free survival rate and disease-specific survival rate were 100%, 91.1% and 100%, respectively. To conclude, SC-HART combined with S-1 for locally advanced rectal cancer was well tolerated and produced good short-term outcomes. SC-HART therefore appeared to have a good feasibility for use in further clinical trials.

## INTRODUCTION

Neoadjuvant radiotherapy (NA-RT) combined with chemotherapy and total mesorectal excision have been adopted as the standard treatment for locally advanced rectal cancer (RC). Two different approaches of NA-RT are commonly in use for RC: short-course NA-RT (25 Gy in five fractions) and long-course NA-RT (45–50.4 Gy in 25–28 fractions) [1–6]. In addition, several hyperfractionated regimens of NA-RT have been reported to have separate early and late radiation effects, with the goal of improving local control while limiting late tissue toxicity [7–15]. However, the best approach for NA-RT remains unclear.

The addition of chemotherapy to preoperative conventional long-term radiotherapy has been demonstrated to be feasible, with enhanced tumoricidal effects [16]. The use of 5-fluorouracil (5-FU)-based chemotherapy has gained widespread acceptance for the treatment of locally advanced rectal adenocarcinoma. S-1 is a novel oral anticancer drug composed of tegafur, 5-chloro-2, 4-dihydroxypyridine, oteracil (which was designed to enhance the oral efficacy of tegafur), and a prodrug of 5-FU. S-1 has been demonstrated to enhance the radiation response of human colon cancer *in vitro* and also in xenograft models [17]. In addition, several clinical studies have shown that NA-RT combined with S-1 had mild toxicity and an equivalent efficacy to that of other regimens of chemoradiotherapy used for RC [18–20]. However, the tolerability and efficacy of NA-RT in an accelerated hyperfractionated regimen combined with S-1 is unclear.

The purpose of the present study was to examine the safety and feasibility of a neoadjuvant protocol involving the short-course hyperfractionated accelerated radiotherapy (SC-HART) combined with S-1 for locally advanced RC.

## MATERIALS AND METHODS

### Patients

A total of 238 patients with RC who underwent surgery at Meiwa hospital (Nishinomiya city, Hyogo, Japan) between March 2008 and May 2012, were reviewed in the present study. Of the 238 patients, 98 were treated with NA-RT followed by radical surgery. 33 patients that received preoperative chemoradiotherapy using different protocols were excluded. To examine the adverse events with more precision the following patients were excluded from this study: three patients with distant metastasis found in preoperative examinations, one patient with peritoneal dissemination and one patient with liver metastasis found in the primary surgery, two patients with cT4 RC that showed a bladder invasion and a uterine inversion, one patient with cT2 RC and one patient with cN0 RC. Therefore, a total of 56 patients with RC of cT3N1M0 located in the lower rectum (Rb) were analyzed in the present study. All NA-RT was performed at a single institution and all surgery was performed by one surgical team. In addition, all patients received consecutive treatment.

Preoperative clinical staging included a clinical assessment, computed tomography (CT) scans between the chest and whole pelvis, full blood analysis including carcinoembryonic antigen, and a colonoscopy with biopsy. Thereafter, all patients were staged according to the TNM classification.

All patients gave informed consent for participation in this study. This study was approved by the Ethics Committee of Hyogo College of Medicine (TCOG GI-0901).

### Preoperative treatment

All eligible patients received SC-HART at the Hospital of Hyogo College of Medicine.

All patients were placed in a supine position and helically scanned on an Aquilion LB (Toshiba, Japan) CT unit. For each patient, a planning CT scan of the entire pelvis from the lower abdomen to below the ischial tuberosities was obtained at 5-mm intervals. The CT dataset was transferred to the FOCUS XiO™ (CMS Inc., St Louis, MO), treatment-planning system to outline the volumes of interest.

The gross target volume (GTV) included the primary rectal tumor and the nodal metastasis. The clinical target volume (CTV) contained the GTV with a 0.5-cm margin, as well as the perirectal, presacral and internal iliac nodes. The planning target volume (PTV) was the CTV with a 0.5-cm margin. Additionally, there was an additional 7-mm leaf margin to the PTV, in order to cover the PTV more homogeneously. Then, the field margins were expanded according to the following protocol.

The field margins of each beam were defined as follows: the cranial margins were the anterior iliac crests or the L4–5 interspace, the caudal margins were the ischial tuberosities, the lateral margins were expanded 1.5 cm beyond the sacroiliac joint, the anterior margins were the dorsal edge of the pubic joint, and the posterior field margins were designed to include the posterior edge of the sacrum.

RT was performed using a 3-D conformal RT technique, which was typically done with a 4-field box technique using 10-MV photons. The planned radiotherapy was delivered using a Mevatron KD2/50 Primus device (Toshiba, Tokyo, Japan) between 2008 and 2009, and an Elekta Synergy device (Elekta, Crawley, UK) from 2009 onward. The patients were treated with a dose of 2.5 Gy twice daily, with an interval of at least 6 h between fractions, up to a total dose of 25 Gy (2.5 Gy × 10 fr). The protocol recommended a treatment time from Monday to Friday. In all patients, S-1 (Taiho Pharmaceutical Co., Tokyo, Japan) was administered orally twice a day in a dose of 80 mg/m^2^ for 10 days combined with SC-HART. Surgery was to be performed between two and three weeks after the end of the radiotherapy. The radical surgery was performed using the technique of the internal anal sphincter resection as described previously [21]. We did not perform prophylactic lateral lymph node dissection in the patients without lateral pelvic lymph node enlargement. Toxicity was assessed according to the Common Terminology Criteria for Adverse Events version 3.0.

### Analysis

The patients' data were recorded on standardized forms and were reviewed. The pathological tumor stage was compared with the clinical stage in each patient. Tumor downstaging was defined as a pathological T Stage (0–4), N stage (from 0–2) and stage grouping (Stage 0–IV) less than the clinical staging prior to the treatment. A pathological tumor response was determined by the presence of pathological downstaging or a pathological complete response (pCR). The data are expressed as the means, with the range in parentheses, unless otherwise indicated. The freedom from disease relapse was calculated considering the local recurrence, distant failure and death due to cancer as an event. Therefore, patients who died from unrelated causes were not considered to be treatment failures.

## RESULTS

The 56 patients included 41 males (73.2%) and 15 females (26.8%). The median patient age was 64.6 (39–85) years. The median follow-up term was 16.3 (range, 2–53) months. No patients required abdominoperineal resection prior to NA-RT. All patients completed NA-RT. The acute adverse events before surgery were related to the gastrointestinal tract in three patients (Grade 2 anorexia with fatigue in one patient, Grade 2 diarrhea and dehydration in one patient, and Grade 3 diarrhea and vomiting in one patient). No patients developed any other hematological or non-hematological toxicities prior to surgery. Of the 56 patients, 55 (98.2%) completed the neoadjuvant therapy, while one patient stopped the chemotherapy because of Grade 3 gastrointestinal toxicity. No Grade 4 toxicity was observed. In addition, there were no unexpected toxicities experienced due to this regimen.

The surgical procedure, postoperative therapy and pathological findings are presented in Table 1. The distal margin of the tumor was beneath the peritoneal reflection in all patients. The distance from the dental line was 3.5 (range, –1.0–8.0) cm. Two patients had no margins to the dental line and one patient had the tumor invading beyond the dental line. No eligible patients received lateral lymph node dissection in the present study. The rate of sphincter-saving resection was 94.6% for all eligible patients. When compared with the clinical staging, downstaging was achieved in 25 patients (44.6%), 39 patients (69.6%) and 45 patients (80.4%) in the T staging, N staging and stage grouping, respectively. Perioperative complications developed in 16 patients (28.6%). Two patients developed two complications each. However, there was no perioperative mortality. Adjuvant chemotherapy was administered after surgery to 43 patients (76.8%). Late toxicity was observed in 21 patients (37.5%). Two different toxicities were recorded in each of three patients. There were five patients with Grade 3 late gastrointestinal complications that required treatment with long intestinal tubes. Late genitourinary complications developed in 12 patients; seven patients had Grade 1, three patients had Grade 2 neurogenic bladder, and two patients had Grade 3 dysuria and required self-catheterization temporarily. There were no Grade 4 late toxicities. None of the patients developed local failure during the follow-up term. The disease-free survival rate was 91.1%, although distant failures were found in five patients (8.9 %) at 5, 9, 11, 13 and 50 months after surgery. The metastatic site was the liver in four patients and the lung in one patient.

**Table 1.** Details and results of the surgical procedure and clinicopathological features

	No. of patients (range or % of total)
Type of rectal surgery	
Intersphincteric resection	39 (69.6)
Double stapling technique	14 (25.0)
Miles' resection	3 (5.4)
Perioperative complications	
Total	16 (28.6)
Pelvic infection	12 (21.4)
Pouch necrosis	2 (3.6)
Rectovaginal fistula	1 (1.8)
Wound infection	1 (1.8)
Crush syndrome	1 (1.8)
Bleeding	1 (1.8)
Post-treatment complications	
Total	21 (37.5)
Gastrointestinal complication	13 (23.2)
≤ Grade 2	8 (14.3)
Grade 3	5 (8.9)
Genitourinary complication	12 (21.4)
≤ Grade 2	10 (17.9)
Grade 3	2 (3.6)
Tumor characteristics	
Distance from anal verge (cm)	4.5 (0.0–9.0)
0.0–5.0	38 (67.9)
>5·0–10.0	18 (32.1)
>10	0 (0.0)
Pathological staging	
T0	2 (3.6)
T1	3 (5.4)
T2	20 (35.7)
T3	30 (53.6)
T4	1 (1.8)
N0	39 (69.6)
N1	13 (23.2)
N2	4 (7.1)
CR	2 (3.6)
I	17 (30.4)
II	20 (35.7)
III a	6 (10.7)
III b	10 (17.9)
III c	1 (1.8)
Adjuvant chemotherapy	
S-1	28 (50)
Capecitabine	4 (7.1)
XELOX	4 (7.1)
UFT/UZEL	4 (7.1)
UFT	2 (3.6)
SOX	1 (1.8)
None	13 (23.2)

XELOX = oxaliplatin and capecitabine, UFT = tegafur-uracil, UZEL = leucovorin, SOX = oxaliplatin combined with S-1.

However, none of the patients developed any local failures. The median disease-free survival and overall survival was 15.1 (2–50) months and 16.3 (2–53) months, respectively. In 13 patients with a follow-up of at least 24 months, the disease-free survival rate at 2 years was 92.3%, the median disease-free survival rate was 35.0 (11–50) months, and the overall survival rate was 38.2 (24–53) months. The disease-specific survival rate was 100.0% during the follow-up-term, although one patient died from pneumonia without any recurrences six months after surgery.

## DISCUSSION

A multimodal approach has become the standard of care for locally advanced resectable RC. In addition, the NA-RT regimen of 25 Gy /5 fractions has become one of the most popular regimens [1–4, 6]. However, the high biological equivalent dose (BED) calculation for hypofractionated regimens raises concern regarding the potential for normal tissue toxicity, therefore there remain concerns that NA-RT as a short-course has the potential to be highly toxic [22]. In contrast, the accelerated hyperfractionated regimen of NA-RT seems to be a treatment regimen with a very favorable risk/benefit ratio [12].

The BED of the protocol of the present study was calculated to compare the different fractionations of radiotherapy. The value of α/β was 10 Gy for the rectal tumor. For tumor effects, the overall treatment time was taken into account using the formula: BED = *nd* [1 + (*d*/α/β)] – γ/α (T – Tk), where *n* is the number of fractions (10 in this case) and *d* is the single fraction dose (2.5 Gy in this case). The BED of the SC-HART protocol was 31.3 Gy. Viani *et al*. have reported that NA-RT with a BED > 30 Gy significantly improved the local control and overall survival in a systematic review of randomized controlled trials [23]. Therefore, the efficacy of SC-HART for RC is expected.

Several hyperfractionated regimens of NA-RT have been reported previously (Table 2) [7–15]. The previous reports have presented the non-inferiority compared to conventional protocols and have described the efficacy for RC. The results of the present study were consisted with their report for the short-term follow-up. An interval of ≥ 6 h between the daily fractions was mandatory to allow for the recovery of normal tissue, and the total treatment time of one week was maintained. The 4-field box technique archived a good coverage of the perirectal space where the possible invisible tumor deposits might exist. In addition, we extended the lateral fields of the beams to the posterior edge of the sacrum in order to cover the whole sacrum in the present study. This field was wider compared to that in the previous reports [4, 5]. However, the presacral region is one of the most prominent sites of local recurrence after multimodal treatment for RC [24]. The wider field of the lateral beams may have benefitted the local control in this study.

**Table 2.** Previous studies of neoadjuvant hyperfractionated radiotherapy

Author	Patients	c-Stage	Tumor site	Radiotherapy	Acute toxicity
Guckenberger *et al.* [7]	108	II 55%, III 45%	Low 43%	29 Gy/10fr	Upper GI Grade 2 0.9%
Ceelen *et al.* [9]	50	II or III	5.8 cm	41.6 Gy/ 26fr	GI 32%, GU 4%, Skin 14%
Marsh Rde W *et al.* [10]	16	T3/4,N0/1M0	3.7 cm	50.4 Gy/ 42fr + capecitabine	Diarrhea 62.5%
Liszka L *et al.* [11]	40	T3NxM0	unknown	42 Gy/28fr	unknown
Widder J *et al.* [12]	184	T3Nx	Low 61%	25 Gy/10fr	unknown
Brooks S *et al.* [13]	20	T3	Low 50%	25 Gy/ 15fr	Lower GI 20%, Upper GI 10%, Skin 20%
(This study)	56	T3N1M0	Low 100%, 4.8 cm	25 Gy/10fr + S-1	GI 5.4%


		Downstaging					
Interval before surgery	Sphincter preservation	T	N	UICC	CR	Adjuvant chemotherapy	DFS
Immediately	74.0%	30.0%	Unknown	Unknown	2.0%	38.0%	76.0% (67 months)
Immediately	Unknown	Unknown	Unknown	Unknown	Unknown	50.0%	64.0% (54 months)
4–6 weeks	68.8%	81.3%	(Tumor and/or nodal)		18.8%	Unknown	66.7% (5 years)
Within 1 week	Unknown	27.5%	Unknown	Unknown	Unknown	Unknown	Unknown
Within 1 week	71.7%	59%	Unknown	Unknown	Unknown	Unknown	75% (2 years)
Immediately	Unknown	35%	Unknown	Unknown	Unknown	Unknown	80% (31 months)
2–3 weeks	94.6%	44.64%	69.64%	80.36%	3.6%	76.8%	91.1% (2 years)


GI = gastrointestinal, GU = genitourinary, CR = complete response, DFS = disease-free survival.

Surgery has been performed immediately following short-course NA-RT, although conventionally, fractionated RT required several weeks in the previous reports [1–4, 6–16, 18–22]. We found that there was a higher downstaging rate, including nodal downstaging, with the delivery of lower doses for a total of 25 Gy combined with S-1. To the best of our knowledge, there have been only a few previous reports that showed the efficacy of NA-RT in hyperfractionated regimens for the downstaging effects [9, 10]. For example, Coucke *et al*. have reported that a delay of more than five days following NA-RT provided a better survival outcome in a retrospective study using a hyperfractionated regimen of RT for a total of 41.6 Gy [14]. In addition, Pettersson, *et al*. reported that delayed surgery following NA-RT showed a downstaging effect [25]. Therefore, the duration of time between NA-RT and surgery may affect the downstaging rate.

The use of 5-FU-based chemotherapy with NA-RT has gained widespread acceptance for the multimodal treatment of locally advanced RC [22]. S-1 is an oral anticancer drug that combines tegafur, a pro-drug of 5-FU, with 5-chloro-2, 4-dihydropyrimidine, and has been reported to enhance the radiation response of various tumors. Sadahiro *et al*. reported the efficacy of S-1combined with NA-RT for RC, and noted milder adverse events with an efficacy seemingly equivalent to the use of capecitabine [18]. In addition, the high tolerance of NA-RT combined with S-1 has recently been reported in multiple clinical trials [18–20]. The present study showed not only a high tolerance of SC-HART in the larger number of patients, but also a high downstaging rate, a high local control rate, a high rate of sphincter preservation, and a low rate of anastomotic leakage. These results suggest that SC-HART with S-1is a valid approach for locally advanced RC, although the surgical technique might improve the sphincter preservation rate [21].

Widder *et al*. have reported on a similar regimen of NA-RT without any concurrent chemotherapy, and reported the disease-free survival rate at two years was 75%, although the local control rate was 96% at four years [12]. In this study we found no patients with local failure, and a high rate of disease-free survival at two years after SC-HART with lower delivered doses of 25 Gy given in combination with S-1. However, 76.8% of eligible patients in this study received postoperative chemotherapy, which might have affected the post-treatment outcomes.

Although the incidence of perioperative complications was either consistent with or lower than that described in previous reports, the incidence of late adverse events due to treatment seemed to be higher than that reported in previous studies [2, 7–9, 12, 13, 24]. However, mild gastrointestinal events and urinary tract symptoms were included in the late adverse events in the present study. In addition, the rate of Grade 3 toxicity was 8.9% and 3.6% in GI and GU, respectively. Lange *et al*. reported that 22.6% of patients with RC experienced an aggravation of incontinence after surgery in a large cohort study [26]. Furthermore, all eligible patients had RC in the lower rectum in this study. The tumor site may affect the incidence of the complications.

We acknowledge that there are several limitations that are associated with this study. In the present study, we presented the short-term outcomes for a limited number of patients with cT3N1M0 RC treated with SC-HART combined with S-1, followed by surgery. We confirmed the feasibility of using SC-HART combined with S-1for locally advanced RC in the present study. Although the follow-up term was not long enough to evaluate the local control or the survival rate, the local control rate was 100.0% in this study. Based on the present study, we believe SC-HART combined with S-1 would have a good efficacy for possible invisible tumor deposits, and would be able to reduce the viability of the tumor to benefit a local control rate. Longer follow-up and a prospective controlled study should be performed in the future in order to clarify the efficacy of SC-HART and the additional benefit of S-1.

## CONCLUSION

We herein presented a novel protocol of neoadjuvant therapy using SC-HART combined with S-1 for locally advanced RC, and reported the short-term outcomes. In the present study, SC-HART was well tolerated and produced excellent short-term outcomes in patients with locally advanced RC.
