# Corrigendum to ‘Neoadjuvant short-course hyperfractionated accelerated radiotherapy (SC-HART) combined with S-1 for locally advanced rectal cancer’^†^

**DOI:** 10.1093/jrr/rru068

**Published:** 2014-07-29

**Authors:** Hiroshi Doi, Naohito Beppu, Soichi Odawara, Masao Tanooka, Yasuhiro Takada, Yasue Niwa, Masayuki Fujiwara, Fumihiko Kimura, Hidenori Yanagi, Naoki Yamanaka, Norihiko Kamikonya, Shozo Hirota

**Affiliations:** 1 Department of Radiology, Hyogo College of Medicine, 1-1, Mukogawa-cho, Nishinomiya City, Hyogo, 663-8501 Japan; 2 Department of Surgery, Meiwa Hospital, 4-31 Agenaruo, Nishinomiya, Hyogo 663-8186, Japan

On page 1119, line 37-38, the following sentence appeared: “This study was approved by the Ethics Committee of Hyogo College of Medicine (TCOG GI-0901)”. The protocol number given here was incorrect and should have been removed, so the sentence should have read: “This study was approved by the Ethics Committee of Hyogo College of Medicine.”

The authors apologise for this error.

